# Gradient-Structured AZ31 Magnesium Alloy: Enhanced Room-Temperature Stretch Formability and Associated Deformation Mechanisms

**DOI:** 10.3390/ma19081566

**Published:** 2026-04-14

**Authors:** Zihuan Hua, Chao He, Lintao Liu, Zhihan Wang, Shengwen Bai, Meng Li, Bin Jiang

**Affiliations:** 1 National Engineering Research Center for Magnesium Alloys, College of Materials Science and Engineering, Chongqing University, Chongqing 400044, China; 2 Mingyue Lake Laboratory, Institute of Lightweight Materials and Engineering, Chongqing 401122, China; 3 College of Materials Science and Engineering, Hubei University of Automotive Technology, Shiyan 442002, China; 20240139@huat.edu.cn; 4 CITIC Dicastal Co., Ltd., Qinhuangdao 066000, China

**Keywords:** magnesium alloy sheet, gradient microstructure, stretch formability

## Abstract

In this study, a gradientstructured (GS) AZ31 Mg alloy sheet with high stretch formability is fabricated using turned bearing extrusion (TBE). The mechanism by which the gradient structure contributes to the improvement in formability is elucidated. The Erichsen index of the GS sheet reaches 5.51 mm, representing an increase of up to 89.3% compared to conventional extruded (CE) sheets. During the Erichsen cupping test, when the coarsegrained (CG) layer of the GS sheet is positioned on the inner side, the large grains promote the activation of deformation twins, thereby effectively enhancing the strain accommodation capacity in the thickness direction. Meanwhile, the finegrained (FG) outer layer effectively suppresses the formation of {10$\overset{-}{1}$1} and {10$\overset{-}{1}$1}-{10$\overset{-}{1}$2} twins, reducing local strain concentration.

## 1. Introduction

Magnesium (Mg) and its alloys have attracted sustained interest as lightweight structural metals because of their low density and high specific strength and stiffness [1,2,3,4,5]. In particular, wrought Mg alloy sheets are regarded as promising candidates for lightweight sheet components, yet their roomtemperature formability remains a critical bottleneck. This limitation is primarily associated with the hexagonal close-packed (HCP) crystal structure, in which a strong basal texture is formed during extrusion and rolling and the restricted activity of independent slip systems leads to pronounced plastic anisotropy and limited strain accommodation [6,7,8,9,10]. As a result, under roomtemperature sheetforming conditions dominated by biaxial stretching, such as cupping and related stretchforming operations, deformation localizes readily and triggers premature cracking, especially along complex strain paths [11]. Therefore, improving the roomtemperature formability of Mg alloy sheets is of great importance.

In recent years, considerable effort has been devoted to improving the formability of Mg alloy sheets by increasing their plasticity, thereby enabling the material to sustain larger strains during forming. The increased plasticity of Mg alloy sheets is commonly achieved either by alloying or by optimizing processing routes. For example, additions of rare-earth elements such as Gd [12], Y [13,14,15], and Er [16,17] have been reported to reduce the relative resistance to the activation of non-basal slip with respect to basal slip, which promotes more plastic deformation. Despite their effectiveness, these alloying strategies usually increase material cost. In parallel, process-based approaches including pre-deformation [18,19], asymmetric extrusion [20], and other advanced methods [21,22] have been developed mainly to weaken the basal texture and thus improve plasticity and formability. However, texture modification alone does not necessarily suppress local strain localization, and cracking can still occur in regions where strain concentrates. From this perspective, it is necessary to consider strategies that simultaneously enhance overall deformability and mitigate local instabilities. Gradient structures provide a practical route because they can redistribute strain through a tailored through-thickness microstructure, reducing the tendency of premature strain localization and fracture.

During forming, Mg alloy sheets often experience a complex through-thickness strain distribution. Introducing heterogeneous structures, including laminated composites [20,23] and gradient-structured sheets [24,25], provides an effective route to improve sheet formability. During the deformation, regions with higher plastic deformability can be arranged to coincide with higher local strain, thereby alleviating strain localization. Wang et al. [23] developed AZ31/Mg-Y laminated composites that exhibited enhanced stretch formability, achieving an Erichsen index of 5.3 mm, higher than that of a single-layer AZ31 sheet at 3.1 mm and an Mg-Y sheet at 4.4 mm. For gradient-structured AZ31, Liu et al. [25] showed that placing the coarse-grained side on the inner surface and the fine-grained (FG) side on the outer surface during bending improved formability. Twinning in the coarse-grained region accommodated inner compression, while the fine-grained region delayed crack initiation, leading to better bendability than homogeneous sheets. Although laminated composites can improve formability, internal heterogeneous interfaces may complicate subsequent secondary forming. In contrast, gradient materials do not encounter this issue. Therefore, the rational use of gradient structures can significantly enhance the formability of Mg alloy sheets.

Many techniques have been developed for the fabrication of gradient materials. Huo et al. [26] successfully fabricated AZ31 sheets with a gradient structure through the thickness using hard-plate rolling technology. Qiang et al. [27] fabricated an aluminum alloy with a gradient microstructure using friction stir processing (FSP). Fang et al. [28] used surface mechanical grinding treatment (SMGT) to produce copper rods with surface gradient structures. The gradient materials fabricated using these methods still exhibit certain limitations in the range of grain-size variation. Moreover, high-heat-input techniques such as additive manufacturing often induce significant residual stresses, which are detrimental to subsequent secondary forming [29]. In contrast, asymmetric extrusion technology can produce sheets with gradient structures along the thickness direction, featuring a wide range of grain-size variation and no heterogeneous interfaces.

During sheet-forming processes, the strain distribution along the normal direction (ND) of the sheet is typically non-uniform [30]. For instance, during bending, the outer side of the sample is generally subjected to tensile stress along the length direction, while the inner side experiences compressive stress, resulting in a gradient strain distribution along the ND of the sample [31]. In the early stage of cupping, the inner side of the sample bears biaxial compressive stress, whereas the outer side is subjected to biaxial tensile stress, and the strain distribution also exhibits a gradient along the ND [32]. FG materials possess both high strength and good ductility, as FG structures not only effectively hinder dislocation slip but also suppress stress concentration [33]. Studies have shown that during cupping forming, twins on the inner side of the sample effectively accommodate strain along the thickness direction, thereby reducing the susceptibility to cracking [23]. Tensile twinning is sensitive to grain size, with a Hall–Petch strengthening coefficient higher than that of dislocation slip [34]. Consequently, fine grains are unfavorable for the activation of tensile twinning, whereas coarse grains facilitate their activation. Moreover, when the grain size is excessively large, stress concentration tends to occur within the sample, increasing the risk of cracking [33]. Based on the above analysis, constructing a gradient structure with fine grains on the outer side and coarse grains on the inner side represents an effective approach to enhance the room-temperature formability of Mg alloy sheets.

In this study, AZ31 magnesium alloy sheets with a through-thickness gradient microstructure in grain size are fabricated by asymmetric extrusion using a turned bearing extrusion (TBE) die, as introduced previously [29]. The microstructure of the extruded sheets is characterized by electron backscatter diffraction (EBSD). Stretch formability is evaluated using the Erichsen cupping test. By combining the cupping response from the Erichsen test with microstructural characterization, the influence of the gradient structure on through-thickness deformation behavior and the evolution of strain localization is discussed, thereby explaining the origin of the improved formability.

## 2. Materials and Methods

The details of the AZ31 alloy are presented in Table 1. In previous studies, a TBE die, which produced by Chongqing unversity in Chongqing, China, was used to fabricate AZ31 sheets with a thickness of 3 mm and a gradient structure (GS) along the ND at an extrusion temperature of 450 °C [24]. The TBE die was a novel asymmetric extrusion die featuring a turned bearing. This die introduced a regular gradient strain distribution along the thickness direction; the effective strain at one surface of the die was 9.46, and it gradually decreased. At the 2.2 mm position, the effective strain reached its minimum value of 5.55. Near another die surface, the effective strain gradually increased again, resulting in a gradient distribution of effective strain along the ND, enabling the dynamic recrystallized grain size near the bearing to be distributed in a regular manner through the sheet thickness, thereby effectively constructing a gradient structure in the Mg alloy sheet. In the GS sheets, the grain size initially increased from 12.4 μm to 81.1 μm, followed by a decrease to 45.6 μm. This method was simple and efficient, requiring no additional alloying elements or post-processing treatments. The resulting GS sheet can directly enhance the formability of AZ31 sheets. From the results of strain distribution and temperature distribution, it can be inferred that the transverse direction (TD)-ND plane exhibits a gradient distribution similar to that of the extrusion direction (ED)-ND plane, while the ED-TD plane is a cross-section of the gradient structure taken along the ND, and therefore shows a uniform size distribution [35]. To obtain a gradient microstructure with a monotonic increase in grain size, the present study adopted the same extrusion route. The extrusion temperature of the TBE die was 450 °C, and the extrusion was performed in a single pass. The region exhibiting a decreasing grain size on the CG side was removed, and the remaining 2 mm thick section was prepared for Erichsen cupping tests. The FG and coarse-grained (CG) sheets with nearly uniform grain sizes of 12.1 μm and 63.2 μm, respectively, were prepared for comparison, denoted as conventional extrusion (CE)-FG and CE-CG sheets. CE-FG and CE-CG sheets were extruded at temperatures of 450 °C and 480 °C, respectively, using an extrusion ratio of 30 and a ram speed of 1.1 mm/s. Since the sheets used in this study were all fabricated by hot extrusion, the high temperature during the extrusion process leads to sufficient dynamic recrystallization, resulting in low residual strain that does not affect the formability of the sheets.

**Figure 1:**
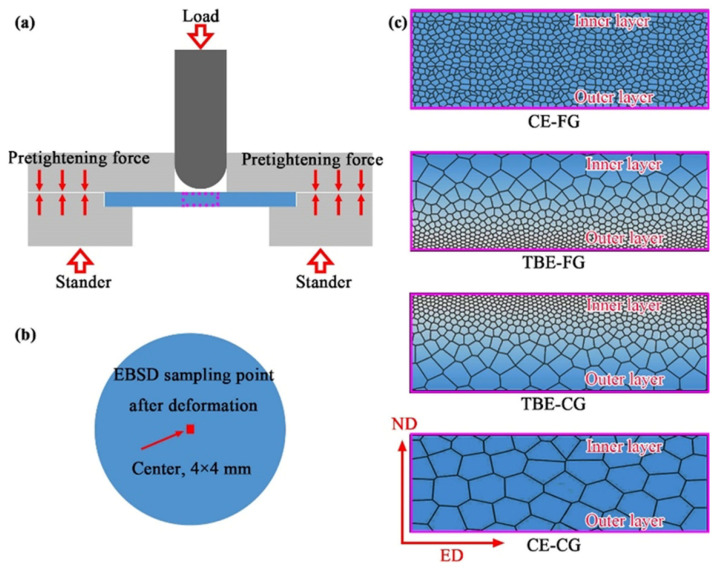
Schematic diagrams of (**a**) Erichsen cupping test, (**b**) sampling method for EBSD and (**c**) arrangements of samples. The four microstructure types correspond to the areas indicated by the dotted square.

The Erichsen cupping test was employed to evaluate the room-temperature (25 °C) stretch formability of sheets, as illustrated in Figure 1. A schematic of the test setup is shown in Figure 1a. The punch featured a hemispherical tip with a diameter of 20 mm, while the die possessed a circular opening with a diameter of 27 mm. Circular sheet specimens, 45 mm in diameter and 2 mm in thickness, were used. During testing, the specimen was clamped between the die and the backing die using bolts. The punch then engaged the specimen surface, applying a forming load. The punch travel at which surface cracking was first observed was recorded as the Erichsen index (IE). The punching speed was 2 mm/s, and graphite grease was used as lubrication. To investigate the influence of gradient microstructures on stretch formability, cupping tests were carried out on four types of samples: CE-FG, TBE-FG, TBE-CG, and CE-CG, as shown in Figure 1c. “CE” and “TBE” denote “conventional extrusion” and “turned bearing extrusion”, respectively, while “FG” and “CG” denote “fine-grained” and “coarse-grained”, respectively. “CE-FG”, “TBE-FG”, “TBE-CG”, and “CE-CG” represent the four microstructures, as shown in Figure 1c. In the TBE process, FG and CG indicate that the fine-grained or coarse-grained regions are positioned on the outer side of the sheet during deformation. All samples were thinned prior to testing. The thinning procedure for the TBE sheets has been described earlier. For the CE sheets, one-sided thinning was applied, resulting in a final thickness of 2 mm. TBE-FG and TBE-CG denote specimens with the FG and CG sides, respectively, oriented outward during testing. A sudden drop in load during the cupping test was taken as the onset of surface cracking on the outer side of the specimen, at which point the test was immediately terminated. To further elucidate the effects of gradient structures on the deformation mechanisms and fracture behavior during stretch forming, additional interrupted Erichsen cupping tests were conducted on all four sample types, stopping the punch travel at 2 mm. The deformed specimens were subsequently extracted and characterized using EBSD. Sampling locations for EBSD analysis are indicated in Figure 1b. The analyzed surface corresponds to the ED-ND plane, where ED refers to the extrusion direction.

The microstructure and texture of the sheets were characterized using EBSD. Prior to the EBSD analysis, the samples were sequentially polished with sandpapers of 400#, 600#, and 1200# grit sizes. This was followed by electrolytic polishing in an AC_2_ solution composed of 1.5 g picric acid, 5 mL acetic acid, and 25 mL ethanol. The electrolytic polishing was performed at −20 °C under a voltage of 20 V, with the current maintained below 0.05 A. The polishing duration was 150 s. During EBSD data acquisition, an accelerating voltage of 20 kV and a beam current of 15 mA were applied. A step size of 2 μm was used. The magnification for the FG region was approximately 500×, while that for the CG region was approximately 100×. The magnification for the entire cross-section was 40×. Upon completion of the EBSD tests, the acquired data were analyzed and processed using EBSD commercial software to extract the final microstructural information.

## 3. Results

### 3.1. Microstructure and Texture

**Figure 2:**
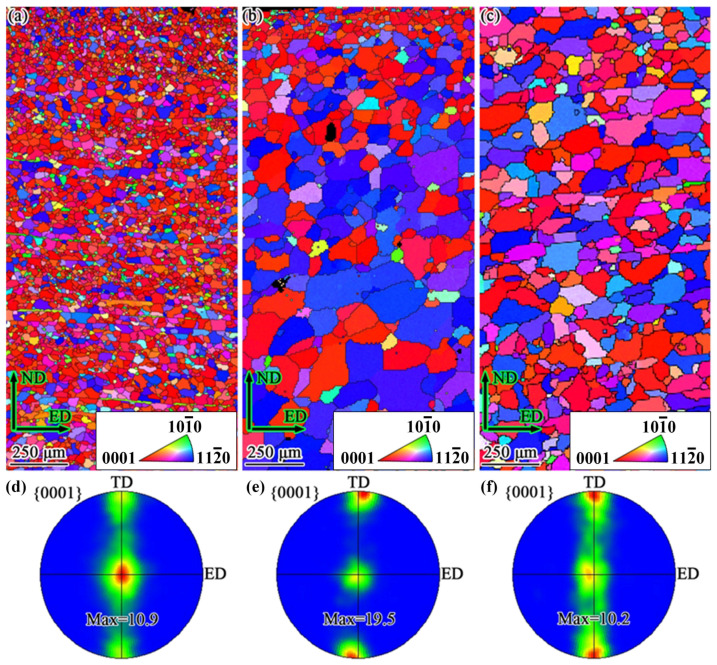
Inverse pole figure (IPF) maps and pole figures (PF) of ED-ND planes, (**a**,**d**) CE-FG sheet, (**b**,**e**) TBE sheet, and (**c**,**f**) CE-CG sheet.

**Figure 3:**
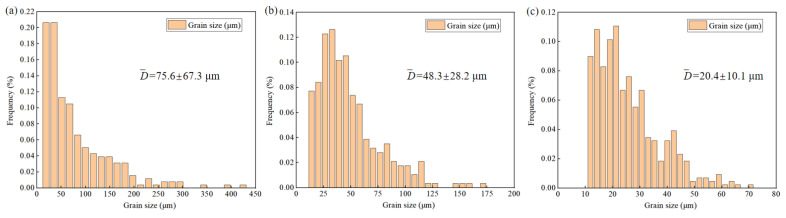
Grain-size histogram: (**a**) CG region, (**b**) transition region and (**c**) FG region.

The microstructures of the CE-FG, TBE, and CE-CG sheets are shown in Figure 2, where dynamic recrystallization is complete in all three samples. The microstructures of the CE-FG and CE-CG sheets are relatively uniform, with average grain sizes of 12.1 μm and 63.2 μm, respectively. In the GS sheet, the grain size increases along the ND from 12.4 μm to 75.6 μm. The detailed statistical results are shown in Figure 3. The textures of the three sheets are also shown in Figure 2, with most of the grain c-axes oriented parallel to the TD-ND plane, resulting in a fiber texture. The maximum relative density (MRD) of the textures for the three sheets is as follows: TBE has an MRD of 19.5, while CE-FG and CE-CG are both close to 10. There are also differences in the MRD positions, with the texture of the CE-FG sheet located at the ND, while the textures of the TBE and CE-CG sheets are mainly concentrated at the TD.

### 3.2. Stretch Formability

**Figure 4:**
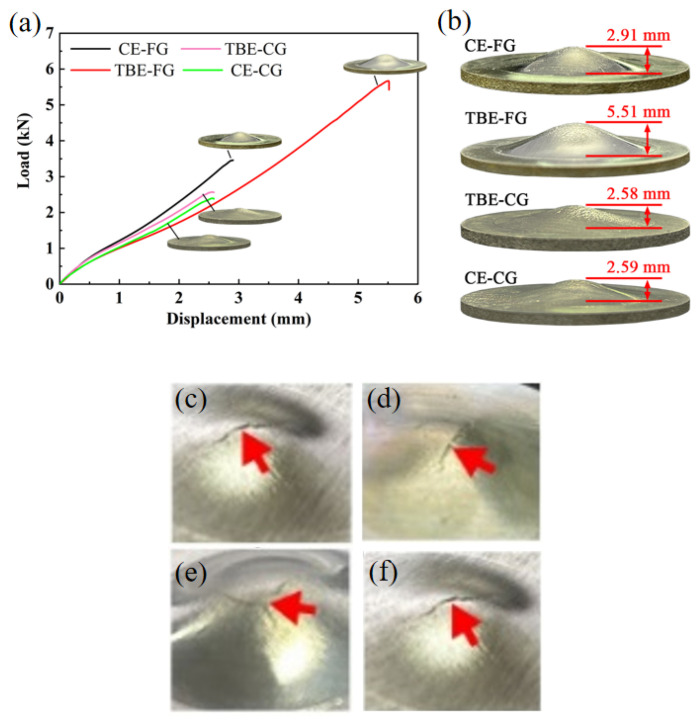
Results of Erichsen cupping tests: (**a**) load–displacement curves, (**b**) samples after stretching and (**c**–**f**) the macroscopic crack morphology of the sample. The arrows indicate macro-cracks.

The load–displacement curves, surface cracking, and IE values for the CE-FG, TBE-FG, TBE-CG, and CE-CG samples during the Erichsen cupping test are shown in Figure 4. During the test, the CE-FG sample exhibits the highest yield load, while the TBE-FG sample has the lowest yield load. Furthermore, under the same punch stroke (before fracture of the CE-CG sample), the CE-FG sample consistently experiences the highest load, whereas the TBE-FG sample consistently experiences the lowest load. This behavior is consistent with the load patterns observed during the bending forming process [24,25]. The TBE-CG and CE-CG samples display low stretch formability, with IE values of 2.58 mm and 2.59 mm, respectively. In contrast, the CE-FG and TBE-FG samples have IE values of 2.91 mm and 5.51 mm, respectively. The detailed results are shown in Table 2. Previous studies have measured the IE value of AZ31 sheets. Here are a few examples. Zhang et al. reported an IE value of 4.82 mm for AZ31 sheets, Han reported an IE value of 5 mm, and Chaudry reported an IE value of 2.2 mm [36,37,38]. Thus, the IE value of 5.51 mm obtained in this study represents a relatively high level.

It is evident that when the fine-grained region of the GS sheet is placed on the outside (TBE-FG sample), the stretch formability of the sheet is significantly improved. Compared to the CE-FG sample, the IE value of the TBE-FG sample increases by 89.3%. A common feature of the CE-CG and TBE-CG samples is that the CG region is located on the outer side. Therefore, it can be inferred that the CG region on the outer side of these two samples plays a crucial role in their fracture behavior during the Erichsen cupping test.

## 4. Discussion

### 4.1. Deformation Mechanism Analysis

**Figure 5:**
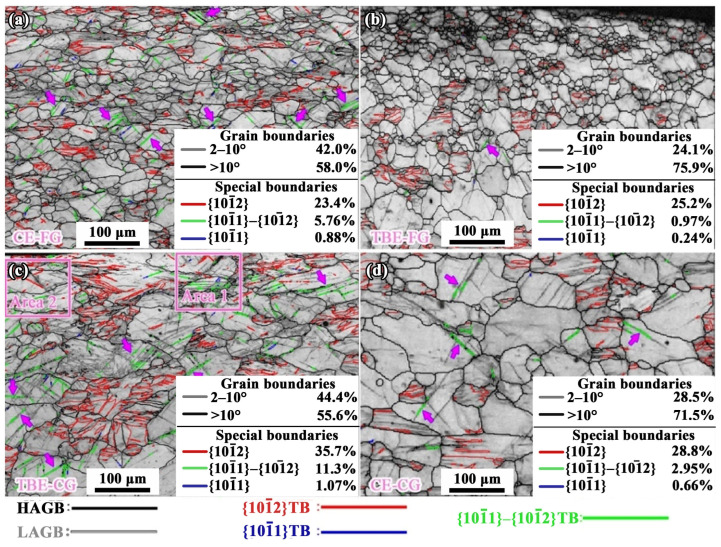
Analyses of grain boundaries at the outer side of stretching samples: (**a**) CE-FG, (**b**) TBE-FG, (**c**) TBE-CG and (**d**) CE-CG samples. The arrows indicate secondary twins.

The results show that the TBE-FG sample exhibits superior stretch formability compared with the other samples. To elucidate the origin of this improvement, the deformation mechanisms of the Mg alloy sheet with a gradient microstructure are systematically analyzed. The EBSD characterization is conducted on the stretched samples with a punch stroke of 2 mm, with the observation plane being the ED-ND plane. EBSD scans are performed at positions close to both the inner and outer surfaces of the sample to reveal the strain accommodation mechanism on both sides and to investigate the fracture mechanism of the sample. The grain boundary analysis of the outer side of the sample is shown in Figure 5, with the proportions of various types of grain boundaries listed in Table 3.

Multiple deformation modes jointly accommodate biaxial tensile straining on the outer surface of the samples. Consistent with the substantial contribution of dislocation slip, all four conditions (CE-FG, TBE-FG, TBE-CG, and CE-CG) exhibit a pronounced fraction of low-angle grain boundaries (*f*_LAGB_), with *f*_LAGB_ of 42.0%, 24.1%, 44.4%, and 28.5%, respectively. Tensile twinning is also prevalent, giving tensile twin-boundary fractions (*f*_TB_) of 23.4%, 25.2%, 35.7%, and 28.8%, respectively. On the outer side, the fractions of secondary twin boundaries (*f*_STB_) are occasionally detected, with *f*_STB_ of 5.76%, 0.97%, 11.3%, and 2.95%, respectively. In contrast, compression twins are rare and only locally present at the outer surface.

It can be observed that the *f*_LAGB_ and *f*_STB_ on the outer side of the TBE-CG sample are the highest, while those of the TBE-FG sample are the lowest. Additionally, the proportion of compression twin boundaries is extremely small, much lower than that of the secondary twin boundaries. This may be due to the complex stress state during the Erichsen cupping process, where compression twins form under localized stress concentrations. After twinning, the crystal orientation of the grains undergoes a 56° rotation. The compression twins, after crystal rotation, facilitate the formation of tensile twins, which appear within the compression twins and eventually consume the original compression twins, forming secondary twins [39].

**Figure 6:**
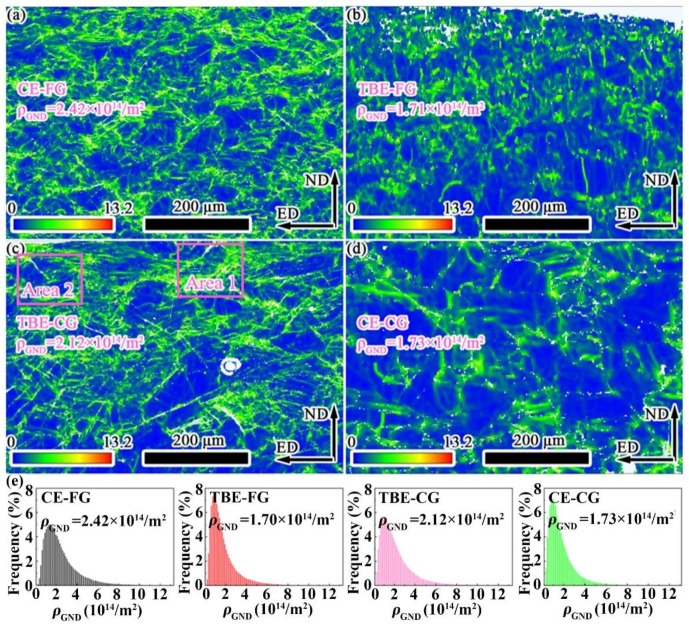
Distribution chart of *ρ*_GND_ at the outer side of stretching samples: (**a**) CE-FG, (**b**) TBE-FG, (**c**) TBE-CG and (**d**) CE-CG samples. (**e**) Frequency distribution of *ρ*_GND_.

The distribution of geometrically necessary dislocations (GND) on the outer side of the samples is shown in Figure 6. The average GND densities on the outer side of the CE-FG, TBE-FG, TBE-CG, and CE-CG samples are 2.42 × 10^14^/m^2^, 1.71 × 10^14^/m^2^, 2.12 × 10^14^/m^2^, and 1.73 × 10^14^/m^2^, respectively. It is evident that both the CE-FG and TBE-FG samples exhibit higher GND densities on the outer side, while the GND densities on the outer side of the TBE-FG and CE-CG samples are relatively lower. Although multiple deformation mechanisms coordinate the deformation on the outer side of the stretched samples, the area fractions of compression twins and secondary twins are very low, and the corresponding strain accommodated by these mechanisms is minimal. Combining the area fractions of twin boundaries, it can be concluded that the outer side of the CE-FG sample experiences the highest biaxial tensile strain, while the outer sides of the TBE-FG and CE-CG samples undergo relatively smaller tensile strains.

It is noteworthy that the locations of secondary twins and compression twins indicated by purple arrows in Figure 5 correspond to areas with relatively high GND densities in Figure 6. This suggests severe strain concentration near these twin boundaries. According to previous studies, the mobility of twin boundaries, particularly those of compression and secondary twins, is very limited. Dislocation motion is blocked when it encounters these boundaries. Unlike tensile twins, which can relieve stress by twin growth, compression and secondary twins cannot effectively alleviate stress, leading to significant stress concentrations [32]. These stress concentrations from the two types of twins can serve as nucleation sites for cracks, as reported in the literature [32]. The boundary analysis of the TBE-CG sample indicates that it has the highest proportion of secondary twin boundaries and compression twin boundaries on its outer side, suggesting that the strain concentration in this sample is the highest among the four groups. From the perspective of strain concentration, it can be concluded that, compared to the bending process, the Erichsen cupping process is more sensitive to local strain concentration. As a result, the TBE-CG and CE-CG samples exhibit better bending formability than the CE-FG sample but poorer stretch formability [24,25].

**Figure 7:**
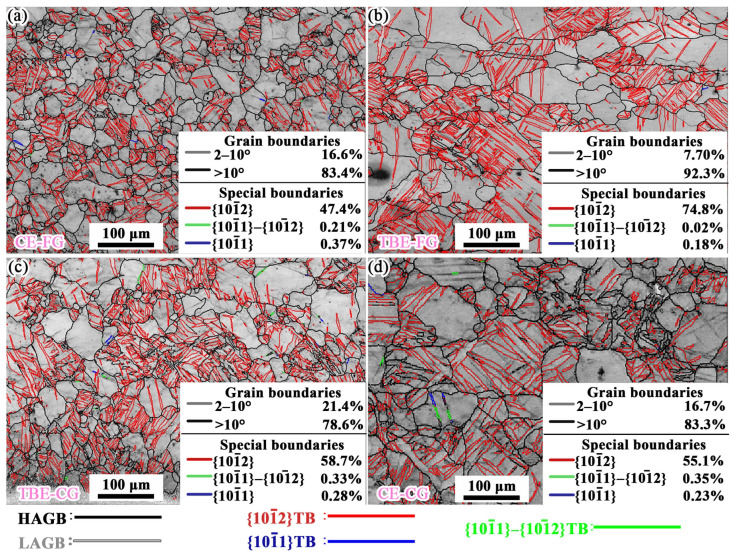
Analyses of grain boundaries at the inner side of stretching samples: (**a**) CE-FG, (**b**) TBE-FG, (**c**) TBE-CG and (**d**) CE-CG samples.

The strain coordination mechanism on the inner side of the stretched samples is illustrated in Figure 7. During the early stage of the Erichsen cupping test, the inner side of the sample experiences biaxial compressive stress, which leads to the formation of a significant number of tensile twins. The fractions of tensile twin boundaries (*f*_TB_) in the CE-FG, TBE-FG, TBE-CG, and CE-CG samples are 47.4%, 74.8%, 58.7%, and 55.1%, respectively. Among all samples, the TBE-FG sample exhibits the highest *f*_TB_ on the inner side. Additionally, there is a certain proportion of LAGB on the inner side of the samples. The *f*_LAGB_ values for the CE-FG, TBE-FG, TBE-CG, and CE-CG samples are 16.6%, 7.69%, 21.4%, and 16.7%, respectively. For all four samples, the fractions of compression twin boundaries and secondary twin boundaries on the inner side are less than 1%, indicating that these two deformation mechanisms contribute negligibly to the strain on the inner side of the samples.

**Figure 8:**
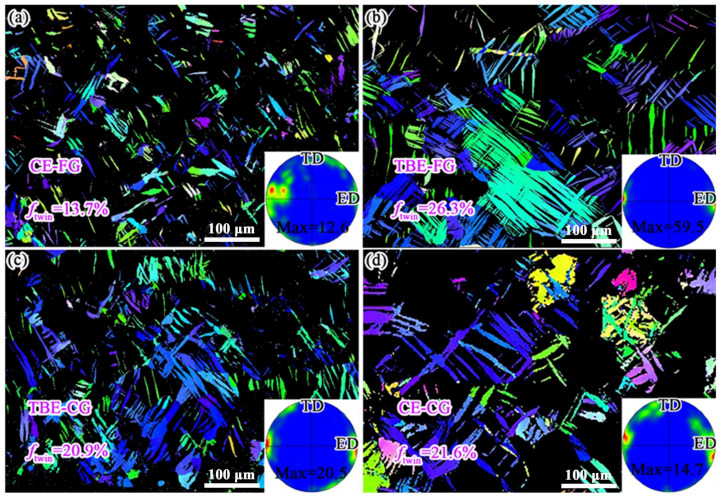
Tensile twins at the inner side of stretching samples extracted from the EBSD data: (**a**) CE-FG, (**b**) TBE-FG, (**c**) TBE-CG and (**d**) CE-CG samples.

**Figure 9:**
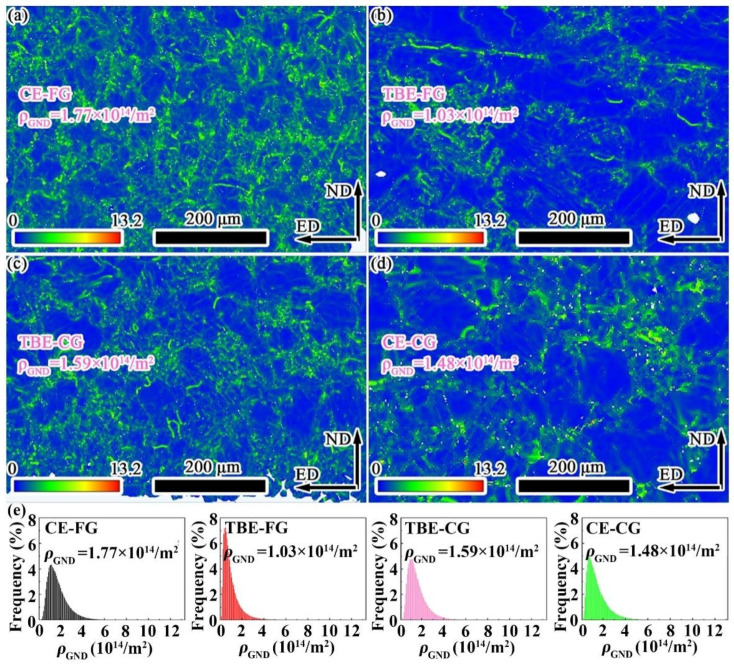
Distribution chart of *ρ*_GND_ at the inner side of stretching samples: (**a**) CE-FG, (**b**) TBE-FG, (**c**) TBE-CG and (**d**) CE-CG samples. (**e**) Frequency distribution chart of *ρ*_GND_.

To quantify the area fraction of tensile twins in the interior of the four samples, twin data were extracted and analyzed from the EBSD results, as shown in Figure 8. The area fractions of tensile twins in the interior of CE-FG, TBE-FG, TBE-CG, and CE-CG samples are 13.7%, 26.3%, 20.9%, and 21.6%, respectively. Among the four samples, the TBE-FG sample exhibits the highest area fraction of tensile twins in the interior, indicating that the strain coordinated by tensile twinning is the greatest in this sample. Additionally, the *ρ*_GND_ in the interior of CE-FG, TBE-FG, TBE-CG, and CE-CG samples are calculated to be 1.77 × 10^14^/m^2^, 1.43 × 10^14^/m^2^, 1.59 × 10^14^/m^2^, and 1.48 × 10^14^/m^2^, respectively, as shown in Figure 9. In the case of the stretched samples, the CE-FG and TBE-CG samples exhibit higher *ρ*_GND_ and lower area fractions of tensile twins in the interior. This is because the grain size in the interior of these two samples is relatively finer, which is less favorable for the formation of tensile twins compared to the TBE-FG and CE-CG samples, leading to a greater contribution from dislocation slip to the total strain [34,40]. Moreover, the *ρ*_GND_ in the interior of the CE-FG and TBE-CG samples is significantly lower than that in the exterior, while the boundary fraction of tensile twins in the interior is much higher than that in the exterior. According to literature reports, the tensile twins formed on the inner surface of the sample during the Erichsen cupping process can effectively coordinate the strain along the thickness direction of the sample. For example, Wang et al. [23] designed an AZ31/Mg-Y composite sheet, where AZ31 was placed on the inner side during the Erichsen cupping process. The stretch formability of this composite sheet even exceeded that of single-layer AZ31 and Mg-Y alloy sheets. This was because the Mg-Y layer on the outer side of the composite sheet had a weaker texture, which provided higher plasticity, while the AZ31 layer on the inner side was more prone to forming tensile twins. These tensile twins helped coordinate the strain along the thickness direction of the sample through the mechanism of twinning. As a result, the stretch formability of the AZ31/Mg-Y composite sheet was improved by 71.0% and 20.5%, compared to single-layer AZ31 and Mg-Y alloy sheets, respectively.

In this study, when the CG layer of the GS sheet is placed on the inner side, the stretch formability of the sheet is significantly improved. The mechanism behind this improvement is similar to the findings from Wang et al. [23] in their study of composite sheets. By comparing the tensile twin area fractions on the inner side and *ρ*_GND_ on the outer side of different stretched samples, it can be observed that increasing the grain size on the inner side promotes the formation of tensile twins. These tensile twins help coordinate the strain along the thickness direction of the stretched sample, thus reducing the local strain concentration on the outer side. This conclusion can be inferred from the relatively low *ρ*_GND_ on the outer side of the TBE-FG and CE-CG samples. From this perspective, increasing the grain size is beneficial for improving the stretch formability of the sheet. However, during the Erichsen cupping process, the biaxial tensile stress on the outer side of the sample is more sensitive to strain concentration. This is because when the grain size is larger, the outer side of the sample is more prone to generating compression and secondary twins. Due to the limited mobility of the boundaries of these twins, they induce significant local strain concentration during subsequent deformation. Therefore, the stretch formability of the TBE-CG and CE-CG samples is worse than that of the CE-FG sample. Based on the above discussion, the effect of grain size on the stretch formability is bidirectional. Therefore, when the CG layer of the GS sheet is placed on the inner side and the FG layer on the outer side during cupping tests (as in the TBE-FG sample), it not only coordinates the strain along the thickness direction of the sheet and reduces the tensile strain on the outer side but also effectively reduces the strain concentration on the outer side. As a result, the stretch formability of the TBE-FG sample is significantly improved compared to the CE-FG sample.

### 4.2. Fracture Mechanism Analysis

**Figure 10:**
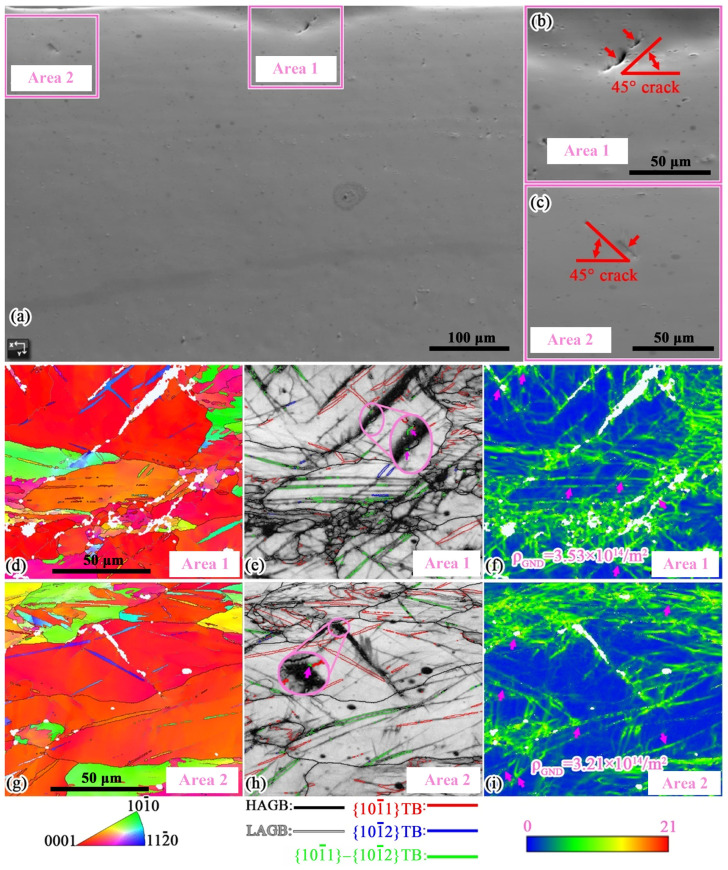
EBSD analyses of Area 1 and Area 2 of the TBE-CG sample: (**a**–**c**) SEM graphs, (**d**–**f**) analyses of Area 1 and (**g**–**i**) analyses of Area 2. The arrow inside the circle indicates secondary twins.

The EBSD analysis of the enlarged regions in Area 1 and Area 2 of Figure 5c is conducted to investigate the crack initiation mechanism, as shown in Figure 10. It can be observed that the angle between these two cracks and the sample surface is 45° in both cases. The angles between the compression and secondary twin boundaries shown in Figure 5 and the sample surface are also concentrated within the range of 45 ± 15°, which is consistent with phenomena reported in the literature [40]. Therefore, it can be inferred that the formation of these two cracks may be attributed to the following reasons: these two types of twins cause significant strain concentration, and the cracks initiate and propagate along the twin boundaries. In the enlarged regions marked by ellipses in Figure 10e,h, it can be observed that both cracks contain a small amount of material that has been torn apart by the crack, and the orientation difference between this material and the adjacent grains is 38°, which corresponds to the characteristic grain boundary angle of secondary twins. This suggests that the crack formation process is as follows: significant strain concentration occurs near the secondary twins, followed by crack initiation at the strain-concentrated location, and the cracks propagate along the secondary twin boundaries, forming longer cracks. In Figure 10f,i, it can be observed that, due to the tearing action of the crack, there is a significant dislocation accumulation at the crack tip, resulting in the *ρ*_GND_ of the crack tips being 3.53 × 10^14^/m^2^ and 3.21 × 10^14^/m^2^, which are much higher than the *ρ*_GND_ on the surface of the TBE-CG sample (2.12 × 10^14^/m^2^). Additionally, the two cracks propagate to the grain boundaries of adjacent grains and then stop. Due to the tearing action of the crack, significant strain concentration appears within the neighboring grains. This indicates that, during the 2 mm stroke of the punch, an initial crack is formed in the TBE-CG sample, but the propagation of this initial crack is temporarily suppressed by the coarse grain boundaries. In the subsequent deformation process, under the effect of strain concentration at the crack tip, these two cracks may tear neighboring grains, leading to the formation of macroscopic cracks on the outer surface of the sample. In conclusion, during the Erichsen cupping process of AZ31 sheet, strain concentration caused by secondary twins and compression twins is the main cause of sample failure.

## 5. Conclusions

In this study, Erichsen cupping tests were conducted on CE-FG, TBE-FG, TBE-CG, and CE-CG AZ31 samples. The results show that when the FG region of the GS sheet is placed on the outer side, the AZ31 sheet exhibits excellent stretch formability. This study also analyzes the deformation mechanisms during the stretch forming of the sheets. The main conclusions are as follows:

The IE values of CE-FG, CE-CG, TBE-FG, and TBE-CG are 2.91 mm, 2.59 mm, 5.51 mm, and 2.58 mm, respectively. Notably, the TBE-FG sheet exhibits an 89.3% improvement in IE value compared to the CE-FG sheet, highlighting the significant enhancement in stretch formability achieved through the gradient structure.

During the Erichsen cupping test, the FG region on the outer side of the TBE-FG sheet suppresses the formation of {10$\overset{-}{1}$1} and {10$\overset{-}{1}$1}-{10$\overset{-}{1}$2} twins. These twin boundaries exhibit low mobility, which tends to induce strain concentration and subsequently lead to crack initiation. Meanwhile, the CG region on the inner side promotes the activation of {10$\overset{-}{1}$2} tensile twins, facilitating strain accommodation along the thickness direction. In contrast, the TBE-CG and CE-CG sheets, both featuring coarse grains in the outer region, are more susceptible to the formation of {10$\overset{-}{1}$1} and {10$\overset{-}{1}$1}-{10$\overset{-}{1}$2} twins. Coarse grains exhibit heightened sensitivity to the resulting strain concentration, making them prone to crack initiation, which accounts for their relatively poor stretch formability. For the CE-FG sheet, the outer FG layer effectively suppresses {10$\overset{-}{1}$1} and {10$\overset{-}{1}$1}-{10$\overset{-}{1}$2} twinning, leading to a more uniform strain distribution and thus superior stretch formability compared to TBE-CG and CE-CG. However, the inner region of the CE-FG sheet is less capable of generating tensile twins to accommodate thickness-direction deformation, resulting in lower stretch formability than that of the TBE-FG sheet.

## Figures and Tables

**Table 1:** Element contents of AZ31.

Element	Al	Zn	Mn	Gd	Zr	Mg
Content (%)	3.05	1.01	0.55	-	-	95.39

**Table 2:** The Erichsen cupping test results of the CE-FG, TBE-FG, TBE-CG, and CE-CG samples.

Sample	CE-FG	CE-CG	TBE-FG	TBE-CG
IE (mm)	2.91 ± 0.14	2.59 ± 0.22	5.51 ± 0.11	2.58 ± 0.18

**Table 3:** Proportion of main grain boundary species and *ρ*_GND_ at the outer side of stretching samples.

Samples	*f*_LAGB_ (%)	*f*_TB_ (%)	*f*_STB_ (%)	$\rho_{\mathrm{GND}}$ (×10^14^/m^2^)
CE-FG	42	23.4	5.76	2.42
TBE-FG	24.1	25.2	0.97	1.71
TBE-CG	44.4	35.7	11.3	2.12
CE-CG	28.5	28.8	2.95	1.73

## Data Availability

The original contributions presented in the study are included in the article, further inquiries can be directed to the corresponding authors.
